# Effects of Early Talent Promotion on Junior and Senior Performance: A Systematic Review and Meta-Analysis

**DOI:** 10.1007/s40279-023-01957-3

**Published:** 2023-11-03

**Authors:** Arne Güllich, Michael Barth

**Affiliations:** 1 Department of Sports Science, RPTU Kaiserslautern-Landau, Erwin-Schrödinger-Straße 57, 67663 Kaiserslautern, Germany; 2 Department of Sport Science, University of Innsbruck, Fürstenweg 185, 6020 Innsbruck, Austria

## Abstract

**Background:**

Does younger involvement in talent promotion programs (TPPs) facilitate the attainment of higher performance levels? This question is the subject of the present meta-analysis. Many national sport systems have established TPPs such as federations’ junior squads (including under-age selection teams) and youth sport academies, and many are making expanding investments in TPPs. TPPs seek to select the most advanced youth high performers at young ages, around puberty or younger, and then strive to further accelerate their performance development. However, studies show 25–55% annual athlete turnover within TPPs. In this context, accelerated biological maturation (puberty, growth spurt), high relative age within one’s birth year, and intensified sport-specific childhood/adolescent practice may boost rapid junior performance, but the effects diminish or are reversed by adulthood. Moreover, expanded opportunity costs and risks (time demands, injury, burnout) imposed on young TPP participants may impair their long-term development and even prematurely terminate their career.

**Objective:**

We aimed to provide robust and generalizable evidence on the effects of early talent promotion on junior and senior performance through a systematic review and meta-analysis.

**Methods:**

A systematic literature search was conducted 18/03–03/04/2023 in SPORTDiscus, ProQuest, PsycINFO, PubMed, Scopus, WorldCat, and Google Scholar. We searched for original studies that compared athletes across defined higher and lower performance levels within defined types of sports, age categories, and sexes, regarding their age at commencement of TPP involvement and reported effect sizes or data needed to compute effects sizes. Mean meta-analytic Cohen’s $\overline{d }$ was computed separately for junior and senior athletes. Quality of evidence was evaluated using the mixed-methods appraisal tool.

**Results:**

The search yielded *k* = 51 effect sizes from *N* = 6233 athletes from a wide range of countries and sports, 82% male and 18% female, from 2009 to 2022. The central finding is that effects on short-term junior performance versus long-term senior performance are opposite, whereby higher-performing junior athletes began TPP involvement at younger ages than lower-performing junior athletes, $\overline{d }$ =  − 0.53. In contrast, higher-performing senior athletes began TPP involvement at older ages than lower-performing senior athletes, $\overline{d }$ = 0.56. The findings are robust across different TPPs (federation’s junior squad/selection team, youth academy), individual and team sports, and performance levels compared (international, national, regional). The quality of primary studies was high.

**Discussion:**

The findings are consistent with recent meta-analytic evidence that participation patterns predicting early junior success versus long-term senior success are opposite (starting age, main-sport and other-sports practice amounts, age to reach performance ‘milestones’). We discuss theoretical and practical implications of potential selection and ‘treatment’ effects of TPPs.

**Conclusions:**

Consistent across different populations, early TPP involvement is positively correlated with short-term junior performance but is negatively correlated with long-term senior performance.

**Supplementary Information:**

The online version contains supplementary material available at 10.1007/s40279-023-01957-3.

## Key Points

**Table:** 

The age of selection for talent promotion programs affects later performance.
Effects on early junior performance and on long-term senior performance are opposite. Higher-performing junior athletes began involvement in talent promotion programs at younger ages than lower-performing junior athletes. In contrast, higher-performing senior athletes began involvement in talent promotion programs at older ages than lower-performing senior athletes.
The findings are robust across different types of talent promotion programs (federation’s junior squads, under-age selection teams, and youth sport academies), individual and team sports, and performance levels compared (international, national, regional).

## Introduction

Does younger involvement in talent promotion programs facilitate the attainment of higher performance levels? This question is the subject of the present meta-analysis.

Many national sport systems around the world have established talent promotion programs (TPPs^1^) at local, regional, and national levels. TPPs are the major “tool” of sport federations to promote the development of their athletes. The TPPs are considered a critical building block of athletes’ pathway towards athletic excellence and a crucial resource of nations in the “global sporting arms race” [1], which has incited nations to make expanding strategic investments in TPPs [1–7].

The most common TPPs include sport federations’ junior squads (including under-age selection teams) and youth sport academies^2^ [3, 7–9, 14–22]. The TPPs provide environments, resources, and interventions to talent-identified youth athletes to foster their performance progress [7, 9, 16, 18, 22–25]. These may include training and competing with other athletes who have a similar performance level; participation in additional high-level competitions; expanded training volume; training camps and clinics; educated high-profile coaches; high-profile facilities and equipment; support staff providing sports medicine, physiotherapy, performance analysis, nutritional counseling, career and lifestyle counseling, psychological support, and academic assistance; school timetables adjusted to the sport schedule; transportation; residency; and financial funding provided to youth athletes.

Many TPPs seek to select the youth athletes who are the most advanced high performers within their age category and, once selected, strive to further accelerate their performance progress via expanded amounts of sport-specific practice and competitions, facilitated by the supportive measures provided [7, 9, 16, 17, 22, 24–26]. They seek to involve talent-identified youth athletes at a young age, typically around puberty or younger, because (1) the common belief is that beginning to foster the youth athlete’s development through the TPP nurture at a younger age will lead to higher subsequent performance; (2) TPPs want to secure themselves the (supposed) young talents, before other TPPs and other sports; and (3) beginning TPPs at a young age enables a long total period of continuous TPP nurture until the anticipated age of peak performance [9, 14–19, 22, 26–30].

In contrast, empirical studies have shown that, across age categories, TPPs deselect considerable numbers of previous TPP members and replace them with new “side-entry” athletes (i.e., athletes who enter a TPP at later stages, after a TPP’s initial stage). This has led to annual athlete turnover of 25–47% among youth sport academies and 28–55% among federations’ junior squads [19, 20, 23, 31–33]. The magnitude of the annual athlete turnover is similar across TPP age categories, implying that the entry age varies among TPP participants, where the number of relatively late “side-entry” athletes accumulates across TPP age categories and thus across increasing TPP stages and levels [16, 18, 23].

In this context, selecting the most advanced youth athletes implies specific selection effects in three regards: many of the most advanced youth athletes within an age category (1) have an accelerated biological maturation (especially early onset of puberty and the growth spurt); (2) have been born early within their birth year (relative age effect); and (3) have already had large amounts of sport-specific practice, with little or no other sports practice, prior to the age of selection [5, 34–43]. Each of these factors is associated with increased childhood/adolescent performance, but not necessarily with increased long-term performance in adulthood because these effects mostly diminish or are even reversed by adulthood [34, 36, 42–49].

Furthermore, TPPs may impose expanded costs and risks on the participants in several regards. For example, in many cases, the TPP involvement implies the youth athlete’s relocation, leaving their family, and changing to a new school. In addition, the expanded time demands associated with TPP involvement (more training and competitions, transit time, training camps and clinics, athlete services) enlarge the youth athlete’s opportunity costs (the lost benefit of forgone other activities, such as time with family, friends, academics, other sports, and hobbies) and the increased practice and competition amounts and cumulative physical load may increase their risks of later overuse injuries, burnout, and dropout [7, 22, 42, 43, 50–61].

In summary, TPPs seek to involve talent-identified youth athletes at very young ages, often around puberty or younger [9, 14–19, 22, 26–30], whereas several empirical findings question some of the premises associated with this strategy [16, 18, 20–24, 31–33, 42–49]. In the present article, we systematically review and synthesize empirical studies that have compared higher-performing versus lower-performing athletes regarding the age they commenced TPP involvement. If younger TPP involvement is associated with higher senior performance, this supports the common belief that beginning TPP nurture at younger ages better promotes the youth athlete’s long-term performance development into adulthood. Such a result would also support present policies of establishing TPPs at very young ages and would imply that the funding they are granted is a good investment. By contrast, if younger TPP involvement is unrelated or negatively correlated with senior performance, this suggests that particularly early TPP nurture does not reliably facilitate long-term performance development. Such a result would question present policies of establishing TPPs at very young ages. In addition, given that funding of early TPPs aims to facilitate future senior successes, the funding of early TPPs in their present form would be a malinvestment of, partly public, money.

## Methods

**Fig. 1:**
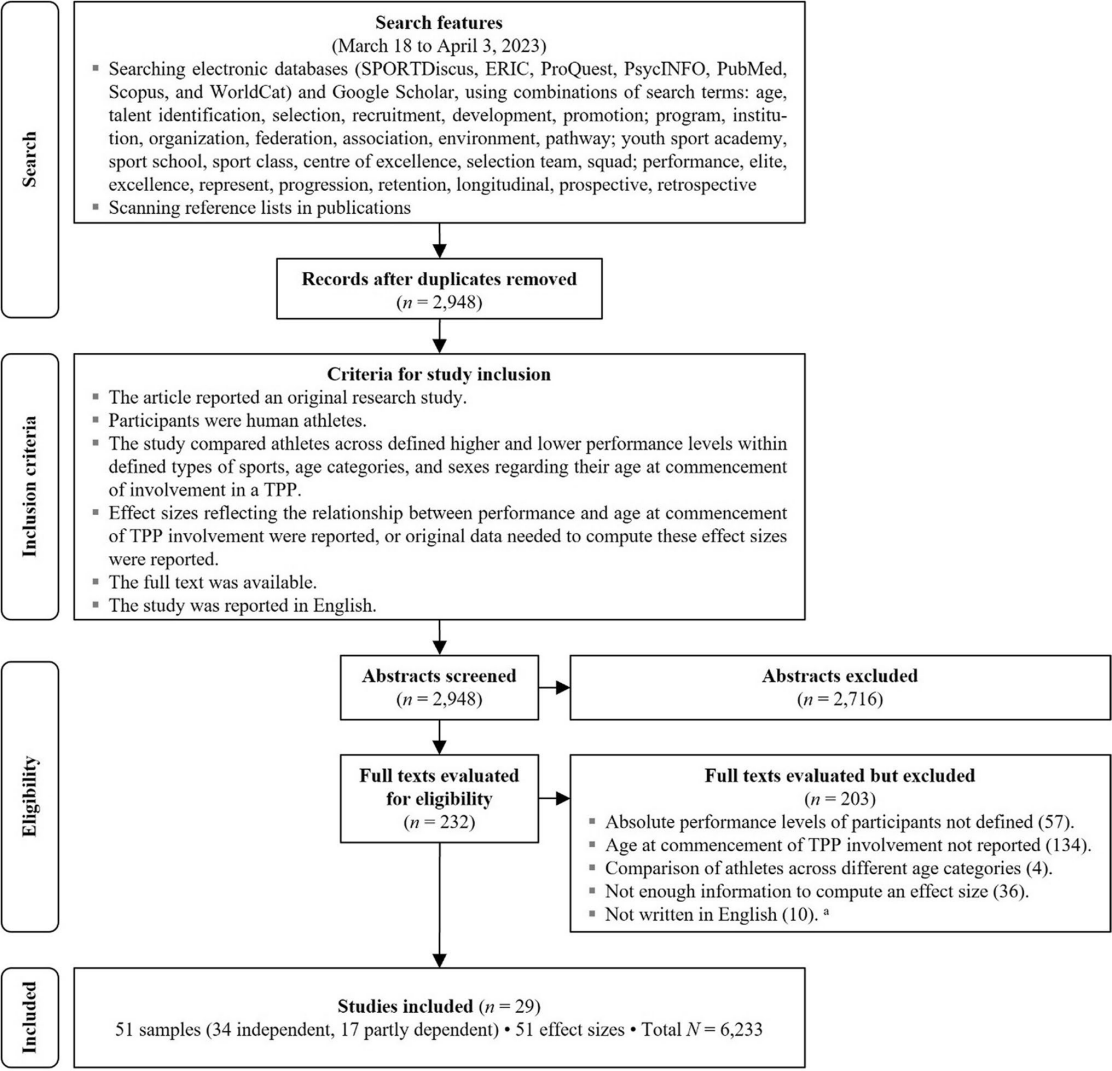
Flow diagram of the literature search and study coding. *TPP* talent promotion program. ^a^Sum > 203 as multiple studies failed to meet several criteria

The study search and selection procedure was guided by the PRISMA 2020 (Preferred Reporting Items for Systematic Reviews and Meta-Analyses [62]) statement. We searched for original studies that (1) compared athletes across defined higher and lower performance levels within defined types of sports, age categories, and sexes, regarding their age of entering a TPP (the federation’s squads or youth sport academies) and (2) reported an effect size reflecting the relationship between performance level and age at commencement of TPP involvement, or original data needed to compute the effect size. Figure 1 shows the flowchart of the major steps of the search and screening, which was conducted from 18 March to 3 April, 2023.

### Sample

The search yielded a total of 51 samples included in 29 study reports from 2009 to 2022. Each study was coded for (1) descriptive data, (2) publication status (published vs unpublished studies, e.g., unpublished theses); (3) sample characteristics (country, sport, sex, age category, performance levels compared); (4) methods of data collection (document analysis or athlete questionnaire or interview); (5) type of TPP (the federation’s squads or youth sport academies); and (6) the effect of the age at commencement of TPP involvement on later performance (see Table S1 in the Electronic Supplementary Material [ESM]).

Table 1 shows characteristics of the total sample. Across studies, all sports of the Olympic Games, as well as cricket, were represented; 68% of the athletes were from team sports (e.g., basketball, cricket, field hockey, handball, ice hockey, soccer, volleyball) and 32% from individual sports (e.g., artistic gymnastics, badminton, fencing, figure skating, judo, race cycling, swimming, tennis, track and field). The participants were from nine countries: Canada, Denmark, Germany, Ireland, Malaysia, the Netherlands, Portugal, Spain, and the UK; 82% were male and 18% female (Table 1).

**Table 1:** Sample characteristics and subsample sizes

	Junior athletes		Senior athletes		Total	
	*k*	*N*	*k*	*N*	*k*	*N*
Overall	16	2328	35	3905	51	6233
Sex^a^						
Male	9	1753	17	3351	26	5104
Female	0	575	4	554	4	1129
Type of talent promotion program^b^						
Federation’s squad/selection team	8	1284	30	3393	38	4677
Youth sport academy	8	1044	5	786	13	1830
Performance levels compared						
World class vs lower	7	1253	23	2675	30	3928
National or regional level vs lower	9	1075	12	1230	21	2305
Types of sports^c^						
Individual sports	6	1061	11	952	17	2013
Team sports	9	1267	20	2953	29	4220
Method of data collection						
Document analysis	3	726	13	2635	16	3361
Athlete questionnaire or interview	13	1602	22	1270	35	2872
Publication status						
Published	7	912	28	2953	35	3865
Unpublished	9	1416	7	952	16	2368

^a^*k* < 16 for juniors and *k* < 35 for seniors, respectively, because 25% of male participants and 91% of female participants were included in 21 samples involving both male and female athletes. These study reports provided the numbers of male and female participants but did not report effects separately for sexes

^b^Total *N* > 6233 because entry age was reported for both federation’s squad and youth sport academy for 274 senior athletes

^c^*k* < 16 for juniors and *k* < 35 for seniors, respectively, because for several multi-sport samples, primary studies did not report effects separately for individual and team sports

#### Talent Promotion Programs

All participants were involved in their federation’s squad or a youth sport academy, or both, at some age. Primary studies reported participants’ age at entry into their federation’s squad for 4677 athletes and age at entry into a youth sport academy for 1830 athletes, thereof, age at entry for both federation’s squad and sport academy for 274 participants (Table 1).

#### Age Categories

We distinguished between junior and senior athletes (athletes competing in the highest, open-age category). This distinction is important because the populations of successful junior athletes and successful senior athletes are not identical but are largely two disparate populations [63]. Junior and senior samples were defined based on the definition of the junior age limit of the international federation for each sport (e.g., female swimming = 17 years; male artistic gymnastics = 18 years; track and field = 19 years). The sample included 37% junior athletes and 63% senior athletes (Table 1).

#### Performance Levels

All junior and senior athletes competed at a regional, national, or international level. We coded the absolute performance level of the samples compared in each study based on athletes’ competition levels: world class (top ten at Olympic Games, world, or continental championships), national class (top ten at national championships or playing in the national premier league), and regional class (below national class, competing at national second tier or lower, regional, province, or state-level championships or leagues).

### Meta-Analytic Approach

All analyses were performed using the publicly available R environment, version 4.3.0. Effect sizes are reported as the standardized mean difference (meta-analytic Cohen’s $\overline{d }$) of the age at commencement of TPP involvement between higher-performing and lower-performing athletes within a type of sport, age category, sex, country, and type of TPP (federation’s squad or youth sport academy). Effects reported as odds ratios in primary studies were converted to Cohen’s *d*. Effect sizes were weighted by the inverse within-study error variance of *d* of each study [64, 65]. Effect sizes ($\overline{d }$ values) of ~ 0.20, ~ 0.50, and ~ 0.80 were considered as small, medium, and large effects, respectively [66]. We searched for outliers, defined as a Cohen’s *d* whose residual had a *z*-score > 3. One outlier was found among the senior samples and was excluded from subsequent analyses ([67], *d* = 3.56). Partly dependent samples were adjusted using Cheung and Chan’s method [68]. All hypothesis testing was two-tailed; a value of *p* < 0.05 was considered statistically significant.

The overall effect of the age at commencement of TPP involvement on later performance was estimated by conducting random effect meta-analyses, separately for junior and senior athletes. Then, mixed-effect models with Wald’s *F* [69] were employed to analyze whether defined subsample characteristics moderated effects. For all moderator analyses, we used the rule of thumb that *k* ≥ 5 is required for each subgroup [70]. We tested for four moderators: (1) age category: junior and senior athletes; (2) type of TPP: federation’s squad and youth sport academy; (3) performance levels: world class versus lower and national or regional class versus lower; and (4) types of sports: individual versus team sports. There were not enough data (*k* < 5) for further moderator analyses across more differentiated categories of sports, single sports, or across sexes.

### Quality Assessment and Risk of Bias

We assessed the quality of the primary studies using the mixed-methods appraisal tool (version 3 [71]). The mixed-methods appraisal tool is a reliable internationally established tool for quality assessment of primary studies in systematic reviews and meta-analyses that provides a dedicated section for quantitative non-randomized studies, as included in this meta-analysis, and has recently been used for similar meta-analyses [63, 72]. We evaluated the primary studies by the two screening questions and the four relevant items for quantitative non-randomized studies within the mixed-methods appraisal tool: representativeness, appropriateness of measurements, completeness of outcome data, and consideration of potential confounders (see Table S2 of the ESM, for further explanation). All studies were assessed by the first author and a random sample of 26 studies (51%) was independently assessed by the second author; inter-rater reliability was excellent (Cohen’s *κ* = 1.00).

In addition, we examined whether studies conducting data collection by document analysis (e.g., official public roster or squad records) versus athlete questionnaires or interviews differed in effect size. Data were collected by document analysis for 54% and by athlete questionnaires or interviews for 46% of the participants. To investigate potential publication bias, we tested whether publication status (published vs unpublished) was a significant moderator and then inspected the funnel plots and computed Egger’s regression analysis.

## Results

The central results are shown in Table 2. Ages at commencement of TPP involvement generally had significant medium-size effects on later performance. However, effects on short-term junior performance and long-term senior performance were opposite (*F* = 63.897, *p* < 0.001), whereby higher-performing junior athletes commenced TPP involvement at *younger* ages than lower-performing junior athletes. In contrast, higher-performing senior athletes commenced TPP involvement at *older* ages than lower-performing senior athletes.

**Table 2:** Effect of age at commencement of involvement in talent promotion programs on short-term junior performance and long-term senior performance^a^

Subsample	$\overline{d }$	95% CI	k	*p* value	*I*^2^
Junior athletes					
Overall	− 0.53	− 0.74 to − 0.32	16	< 0.001	74.94
Talent promotion programs					
Age at entry into federation’s squad^b^	− 0.64	− 0.76 to − 0.52	8	< 0.001	3.55
Age at entry into youth sport academy^b^	− 0.42	− 0.83 to − 0.01	8	0.044	82.15
Performance levels					
World-class versus lower^c^	− 0.63	− 0.76 to − 0.49	7	< 0.001	13.97
National or regional class vs lower^c^	− 0.46	− 0.84 to − 0.09	9	0.016	79.57
Types of sports					
Individual sports	− 0.59	− 0.78 to − 0.40	6	< 0.001	36.11
Team sports	− 0.46	− 0.84 to − 0.09	9	0.016	79.57
Senior athletes					
Overall	0.56	0.40–0.72	34	< 0.001	74.83
Talent promotion programs					
Age at entry into federation’s squad^b^	0.57	0.39–0.74	29	< 0.001	75.50
Age at entry into youth sport academy^b^	0.53	0.13–0.93	5	0.009	71.15
Performance levels					
World-class vs lower^c^	0.65	0.44–0.85	23	< 0.001	73.98
National or regional class vs lower^c^	0.42	0.19–0.65	11	< 0.001	66.58
Types of sports					
Individual sports	0.50	0.33–0.66	11	< 0.001	12.02
Team sports	0.57	0.35–0.79	20	< 0.001	81.11

*CI* confidence interval. The forest plots for junior and senior samples are shown in the Figs. S1 and S2 of the ESM

^a^Note the sign of effects: a positive effect indicates that higher performance was associated with older age at commencement of TPP involvement. A negative effect indicates that higher performance was associated with younger age at commencement of TPP involvement

^b^Involving all performance levels

^c^Involving ages at entry into federation’s squads and youth sport academies

### Moderator Analyses

Effect sizes did not differ significantly between federations’ squads versus youth sport academies (junior sample: *F* = 0.615, *p* = 0.446; senior sample: *F* = 0.024, *p* = 0.877), performance levels compared (junior sample: *F* = 0.285, *p* = 0.602; senior sample: *F* = 1.958, *p* = 0.171), or individual versus team sports (junior sample: *F* = 0.162, *p* = 0.694; senior sample: *F* = 0.002, *p* = 0.966).

There were not enough data for moderator analyses across single sports. Yet, descriptive data from the analytical categories of sports defined in Güllich et al. [42] suggest consistent results across different types of sports: cgs sports (performance is measured in centimeters, grams, or seconds, e.g., athletics, swimming, race cycling, rowing) juniors $\overline{d }$ = –0.56, seniors $\overline{d }$ = 0.36; game sports (e.g., basketball, soccer, rugby, tennis, cricket) juniors $\overline{d }$ = –0.49, seniors $\overline{d }$ = 0.55; combat sports (e.g., judo, wrestling, fencing, taekwondo) juniors $\overline{d }$ = –0.58, seniors $\overline{d }$ = 0.51; artistic composition sports (e.g., artistic gymnastics, rhythmic gymnastics, figure skating, platform diving) juniors $\overline{d }$ = –0.25, seniors $\overline{d }$ = 1.08; and within the largest sport-specific subsample, soccer, juniors $\overline{d }$ = − 0.53, and seniors $\overline{d }$ = 0.57 (see Table S1 of the ESM for *k* and *N* values).

### Quality of Primary Studies and Risk of Bias

All primary studies had a high methodological quality and risk of bias was generally low (see Table S2 in the ESM). Effects did not significantly differ between studies using document analyses versus athlete questionnaires or interviews for data collection (*F* = 2.584, *p* = 0.118).

Effect sizes also did not significantly differ between published and unpublished studies (junior sample: *F* = 3.556, *p* = 0.080; senior sample: *F* = 0.758, *p* = 0.390). The funnel plots were widely symmetrical (see Figs. S3 and S4 in the ESM), and Egger’s regression was non-significant (*b* = 0.88, 95% confidence interval 0.52–1.25, *p* = 0.149).

## Discussion

The study investigated the association of the age at commencement of TPP involvement with later performance. The central finding is that athletes’ age at commencement of TPP involvement had opposite effects on short-term junior performance and long-term senior performance. Higher-performing junior athletes commenced TPP involvement at *younger* ages than lower-performing junior athletes. In contrast, higher-performing senior athletes commenced TPP involvement at *older* ages than lower-performing senior athletes. The findings were robust, in terms of both direction and scale of effects, across federations’ squads and youth sport academies, performance levels compared (international, national, regional), as well as different types of sports.

The results are consistent with recent meta-analytical findings [42, 43, 63]: (1) successful junior athletes and successful senior athletes are not one identical population but are largely two disparate populations [63]. Most successful junior athletes achieve lower competition levels when they are seniors, while most successful senior athletes had achieved lower competition levels when they were juniors. The overlap of successful juniors and successful seniors is the smaller the higher the performance level and the younger the junior age category. (2) Concerning athletes’ participation patterns, several predictors of early junior performance and of long-term senior performance are opposite [42, 43]. Higher-performing junior athletes, compared with lower-performing juniors, started playing their respective main sport at younger ages, accumulated greater amounts of organized coach-led practice in their main sport and less practice in other sports, and achieved performance-related developmental ‘milestones’ at younger ages (e.g., first state, national, or international championships). In contrast, senior world-class athletes, compared with lower-performing senior national-class counterparts, started playing their main sport at older ages, accumulated less main-sport practice and more other-sports practice, and reached performance ‘milestones’ at older ages. It is important to note that across primary studies, the performance-related effects of the different predictors—main-sport starting age, amount of main-sport practice, amount of other-sports practice, and age of ‘milestone’ achievement—were closely correlated with one another (0.63 <|*r*_*s*_|< 0.80) [43].

The findings do not call into question the importance of multi-year sport-specific practice, childhood/adolescent performance development, and their support through TPP nurture. All the senior world-class athletes, senior national-class athletes, and high-performing junior athletes engaged in considerable sport-specific practice over multiple years [42, 43]; many had remarkable performance progress during junior age categories (achieving regional, national, and international junior championships [63]); and all were selected for a TPP at some age. However, a particularly accelerated childhood/adolescent development—typically via an early start, extensive main-sport practice, little or no other-sports practice, and early TPP involvement—is frequent among the highest-performing junior athletes but is rare among the highest-performing senior athletes ([42, 43], the present findings).

### Theoretical Implications

The present meta-analysis complements a recent series of meta-analyses [42, 43, 63, 72, 73] that empirically tested the validity of the ‘hard core of assumptions’ [74] of traditional theories of giftedness and expertise [26, 75–80]. As mentioned above, the assumed premises (1) that achieving a high performance level in childhood/adolescence is a prerequisite for the long-term attainment of a high level of eventual senior performance, (2) that starting sport-specific practice at a younger age leads to a higher level of eventual senior performance, (3) that accumulating a larger amount of organized coach-led main-sport practice leads to a higher level of eventual senior performance, and (4) that accelerated childhood/adolescent performance progress leads to a higher level of eventual senior performance, have all been revealed to be at odds with the empirical evidence [42, 43, 63, 72, 73]. Likewise, the present meta-analysis empirically counters the assumption (5) that younger TPP involvement leads to a higher level of eventual senior performance. Taking the present study and recent evidence together [42, 43], predictors of rapid junior performance and of long-term senior performance are opposite in five aspects: starting age; amount of coach-led main-sport practice; amount of coach-led other-sports practice; age at commencement of TPP involvement; and age of ‘milestone’ achievement.

**Fig. 2:**
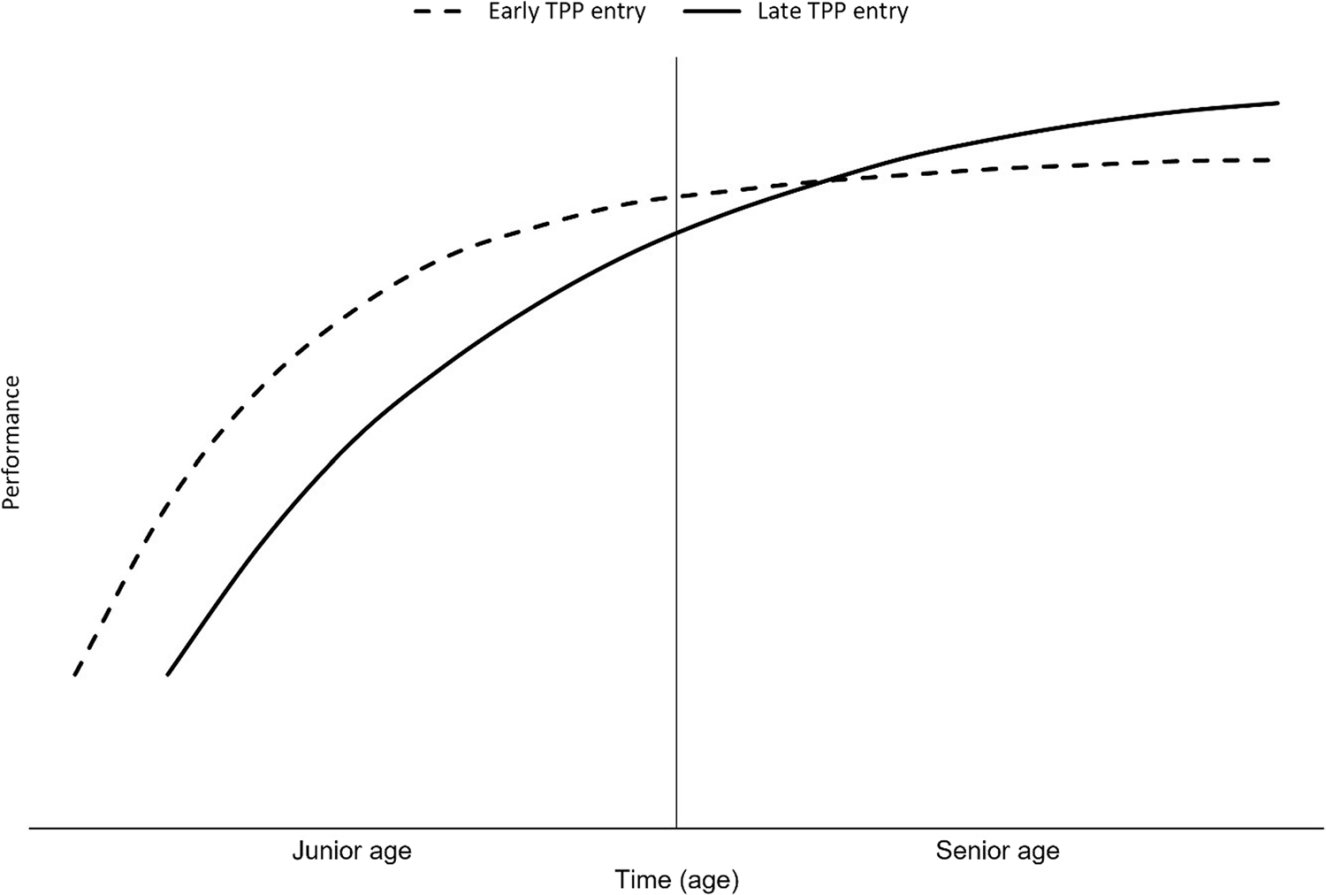
Typical performance development of athletes who commence talent promotion program (TPP) involvement at younger versus older ages. Schematic illustration based on data of the present meta-analysis and [42, 43, 63]. The vertical *Y-axis* symbolizes the junior age limit. Early TPP entry is typically associated with early start to play one’s main sport, extensive specialized main-sport practice, little or no other-sports practice, and early achievement of performance ‘milestones.’ Late TPP entry is typically associated with later start to play one’s main sport, reduced main-sport practice, more other-sports practice, and older ‘milestone’ achievement

To illustrate for this discussion, typical performance trajectories of athletes who commence TPP involvement at younger versus older ages are schematically depicted in Fig. 2. Generally, TPPs include the selection of youth athletes and their ‘treatment’ by applying TPP measures to them. The present findings may thus be attributable to specific selection or intervention effects of TPPs, or an interplay of both. Many of the youth athletes selected at a young age have an early biological maturation (onset of puberty and growth spurt), are relatively old within their birth year, and have previously accumulated large amounts of specialized main-sport practice, with little or no other-sports practice [34–43]. When accelerated early performance progress rests on these factors, this early progress is often associated with reduced long-term *sustainability*, in that the performance trajectory subsequently flattens (Fig. 2) [34, 36, 42–49].

Once selected, the TPPs seek to further accelerate the youth athlete’s childhood/adolescent performance progress via expanded sport-specific practice and competitions, supported by corresponding environments, resources, and interventions [7, 9, 16, 22–25]. The strategy may further boost the youth athlete’s current rate of performance progress and lead to increased short-term performance during junior age categories (Fig. 2). However, it may further compromise long-term sustainability because the large amount of childhood/adolescent specialized practice leads to reduced *efficiency of practice* in subsequent years—the larger the amount of previously accumulated specialized practice the smaller generally the subsequent performance gain per added practice [42, 43]. In economic terms, these athletes’ diminishing return (Gossen’s first law: diminishing performance improvement per added practice amount [81]) is more pronounced than among peers who had only moderate childhood/adolescent main-sport practice combined with other-sports practice and competitions. The data of Barth et al. [43] have shown that senior world-class athletes began to have an advantage in efficiency of practice over senior national-class counterparts in late adolescence and this advantage in efficiency of practice peaked in athletes’ early 20s.

Furthermore, the expanded time demands, and cumulative physical load associated with early TPP involvement, impose additional opportunity costs on the youth athlete and increase their risks of later occurrences that hinder or end the athletic career (e.g., conflicting time demands from sport and school, declining academic performance, injury, burnout, dropout) [7, 9, 50–61, 82, 83].

In contrast, many eventual senior world-class athletes developed outside the TPPs, just based on the youth sport program of their home/local sport club or school, until older ages, thus remaining unaffected by potential dysfunctional effects of early TPP involvement. Senior world-class athletes’ childhood/adolescent *investment pattern* was typically characterized by reduced intensity (less main-sport practice), diversification of investments (two to three sports), greater personal, private contribution (later TPP support), longer total period of “product development” and longer deferral of reward (later ‘milestone’ achievement), reduced total childhood/adolescent costs and long-term risks (injury, burnout, dropout), yielding increased long-term benefit in terms of senior performance (see [42, 43, 84], which also include further in-depth discussion of explanatory hypotheses).

### Practical Implications

Taken together, the recent and present evidence suggests several clear practical implications.

- Given the negative correlation between early TPP involvement and long-term senior performance, the selection age of many TPPs may generally be postponed to older ages.
- Just the youth athlete’s current performance level, be it performance in competitions or in standardized tests of physical and perceptual-motor performance, does not reflect their future potential and is therefore not a sensible selection criterion [16, 18, 34, 63, 72, 85, 86]. Above that, selecting by current performance may have a dysfunctional ‘radiating’ effect [31, 63], in that it stimulates all those seeking admission to TPPs, youth athletes, coaches, parents, and perhaps other stakeholders, to attempt to reinforce early acceleration of the youth athlete’s development prior to the selection age. Instead, provided long-term positive effects of a TPP have been proven, selectors should strive to identify and estimate indicators of the youth athlete’s *potential* for long-term development. In this context, a participation history of moderate main-sport practice while playing other sports over multiple years is a predictor of long-term potential and should be considered for talent selection, while also taking into account whether athletes were born earlier or later within their birth year and whether they have an earlier or later onset of puberty and growth spurt.
- Relatedly, evaluations of TPPs, which often form the basis for their external funding, should not focus on participants’ current junior performance or their short-term progress, including participants’ progression to a subsequent TPP stage or level. This would elicit dysfunctional incentives in that it would further stimulate attempts to select the most advanced youth athletes and then further accelerate their current performance. Rather, evaluations of TPPs should consider their long-term sustainability and focus on the participants’ performance development through subsequent years into adulthood and the long-term senior performance level they eventually achieve, while considering their costs and risks.
- Finally, TPPs should generally seek to enhance the youth athlete’s short-term and long-term benefits while limiting their costs and risks. Specifically, TPPs should limit additional opportunity costs in terms of time demands and risks in terms of amounts of practice and competitions and associated cumulative physical load imposed on the youth athlete.

### Methodological Considerations and Future Directions

The study had several strengths, such as a large international sample including a broad range of individual and team sports; considering the most common types of TPPs; distinguishing short-term effects on early junior performance and long-term effects on later senior performance; comparing higher-performing and lower-performing athletes across regional, national, and international levels; comparing athletes within the same age category, type of sport, sex, country, and regarding age at entry into the same TPP, respectively; and a high quality of the primary studies. Nevertheless, several limitations should be acknowledged: (1) the correlational design of the primary studies does not allow for causal conclusions; (2) most athletes were male, from Olympic sports, and from Western industrialized countries (but not from the largest Western country, the USA). Effects of early TPP involvement may differ in Paralympic sports or in different sport systems such as the USA, developing countries, Eastern Europe, China, or Russia. (3) All athletes competed at a regional, national, or international level, which may imply restriction of range. Effects of early TPP involvement may differ among lower-level or more heterogeneous populations. (4) There were not enough data for moderator analyses across single sports. Effects may vary between single sports. (5) Successful senior athletes who were not selected for a TPP during junior age categories may not have been considered in primary studies. (6) The synthesized primary studies used linear methods of data analysis and did not consider non-linear analyses. Finally, although we used multiple databases, as in any systematic review, bias of availability, country, and language is possible.

A goal for future research is to investigate the extent to which opposite effects of the age at commencement of TPP involvement on short-term and long-term performance are due to specific selection or intervention effects of TPPs, or an interplay of both. That research should examine the effects of the individual and combined TPP measures applied to participants on their short-term and long-term performance, while also considering effects on other short-term and long-term outcomes such as athletes’ health, psychosocial well-being, academic achievements, and persistent sport engagement. Furthermore, researchers should seek to extend investigations into TPPs to populations that are under-represented in present research, especially female athletes, more sport-specific samples, in particular from Paralympic and non-Olympic sports, and samples from more countries, in particular the USA, developing countries, East European countries, China, and Russia. In this context, the present findings indicate that long-term TPP effects on eventual senior performance (and perhaps other long-term outcomes) cannot be inferred by extrapolating findings from junior samples [7, 19, 61, 87, 88]. Rather, relevant statements require comparison of higher-performing and lower-performing senior athletes regarding the TPP environments, resources, and intervention measures they were provided during childhood and adolescence.

It is also interesting to note that multiple decades of extensive research into talent *identification* (TID) [5, 15, 27, 29, 89–99] is contrasted by lacking research into the *purpose* TID is done for: i.e., the effects of the TPP interventions applied to the talent-identified TPP participants [16, 22, 23]. The practical use of further expansion of applied research into early TID may be questionable: First, the long-term prognostic validity of the talent indicators worked out through six decades of research has remained poor [5, 22, 27, 34, 94, 97]. Second, because of the generally low “base rate” (the proportion of youth athletes within the population of interest who eventually become successful senior athletes) [18, 22, 100, 101], even substantial improvement of the predictive accuracy of TID would only yield minimal improvement of the “hit rate” of talent selection procedures in practice (the proportion of selected youth athletes who later become successful senior athletes) [see [22] for an exemplary calculation based on empirical data]. Third, perhaps most importantly, early TPP involvement, for which early TID is done, is negatively correlated with long-term senior performance.

In a broader context, it is apparent that there is no single factor that “makes a champion,” but that talent development is multi-factorial, calling for more investigations considering multivariable interactive predictor effects (e.g., [85, 102]). Furthermore, many factors are likely non-linearly related with performance. For example, eventual senior performance is supposedly a parabolic function of several childhood/adolescent predictors: too early or too late start; too much or too little main-sport practice; too much or too little other-sports practice; and too early or too late TPP involvement are supposedly associated with reduced later performance, while there is presumably some respective optimum in between that is associated with increased later performance. Exploring those optima calls for more non-linear multivariable analyses of factors such as machine learning approaches, logistic regressions, or cluster analyses (for recent examples see e.g., [103–107]).

Finally, the economic concepts of *efficiency*—performance improvement per magnitude of investment, for example, in terms of amounts of practice and TPP measures and resources*—*and *sustainability*—considering the benefit/cost × risk ratio of TPP involvement at shorter and longer terms*—*generally provide a fruitful heuristic for research into talent development [42, 43, 84, 108] because (1) resources are limited (the athlete’s time, body, load tolerance, and health; coaching and facilities; TPP measures); (2) athletes, coaches, and parents seek to expand benefits for the youth athlete while limiting their costs and risks; and (3) effects of several factors vary and may even be opposite regarding short-term versus long-term outcomes. The concepts of efficiency and sustainability apply to research into TPPs as well as other programs such as youth sport programs in general; coaching; athletes’ participation patterns; the ‘microstructure’ of practice; and athlete services such as physiotherapy, nutritional counseling, or psychological support, and lead to three critical research questions [84]:

1. What short-term and long-term costs, risks, and benefits does a program (or do different programs) yield, and to what magnitude and probability?
2. What objective and subjective, material and immaterial value does each of the costs, risks, and benefits have?
3. What is the eventual ratio of the summed value of all benefits relative to the summed value of all costs and risks emerging from a program (or from different programs)?

## Conclusions

Early TPP involvement is positively correlated with short-term junior performance, but is negatively correlated with long-term senior performance. That is, higher-performing senior athletes have developed outside TPPs until older ages than lower-performing senior athletes. The finding questions the practical use of early TPPs and thus of early TID. Research into the effects of individual and combined TPP measures may advance our understanding of the way of functioning of TPPs. That research should consider youth athletes’ short-term and long-term costs, risks, and benefits of TPP participation. For this aim, the economic concepts of efficiency and sustainability provide a fruitful heuristic.

### Supplementary Information

Below is the link to the electronic supplementary material.

Supplementary file1 (DOCX 1139 KB)

## Footnotes

1 Some scholars and practitioners have labeled these programs “talent development” programs. We refrain from using this term because the development of talents takes place both within and outside these programs. Furthermore, some have used “talent identification” programs as an umbrella term referring to all the processes of searching, identifying, selecting, and promoting talents. We suggest the central purpose of these programs is to promote talent development, where talent identification is instrumental to talent selection and selection is instrumental to talent promotion.

2 Youth sport academies have been labeled “elite sport schools,“ “top sport schools,” “talent schools,” and “elite sports classes” in some countries [7–13].

## Declarations

### Availability of data and material

The original data are freely available at https://osf.io/k5xp3/.
