# Supplementary material for: Effects of Early Talent Promotion on Junior and Senior Performance: A Systematic Review and Meta-Analysis
Source: Sports Med. 2023 Nov 3;54(3):697–710. doi: 10.1007/s40279-023-01957-3 (PMC10978645; doi:10.1007/s40279-023-01957-3)
Supplement: Supplementary file 1 — Supplementary file1 (DOCX 1139 KB) [file 40279_2023_1957_MOESM1_ESM.docx]

**Effects of early talent promotion on junior and senior performance:**

**A systematic review and meta-analysis**

**Sports Medicine**

**Electronic Supplementary Material**

Arne Güllich^(a)*^, Michael Barth^(b)^

Running head: Talent promotion and performance

^(a)^ Department of Sports Science

RPTU Kaiserslautern-Landau

Erwin-Schrödinger-Straße 57

67663 Kaiserslautern

Germany

* Correspondence to Arne Güllich: [guellich@sowi.uni-kl.de](mailto:guellich@sowi.uni-kl.de)

^(b)^ Department of Sport Science

Universität Innsbruck

Fürstenweg 185

6020 Innsbruck

Austria

ORCID ID

Arne Güllich <http://orcid.org/0000-0001-6911-2236>

Michael Barth <http://orcid.org/0000-0001-5333-3122>

**Studies in the meta-analysis**

Ford PR, Ward P, Hodges NJ, Williams AM. The role of deliberate practice and play in career progression in sport: the early engagement hypothesis. High Ability Studies 2009;20(1):65-75.

Ford PR, Low J, McRobert AP, Williams AM. Developmental activities that contribute to high or low performance by elite cricket batters when recognizing type of delivery from bowlers’ advanced postural cues. Journal of Sport & Exercise Psychology 2010;32:638-54.

Güllich A, Emrich E. Elite sport and education in Europe. Brussels: European Commission; 2010.

Moesch K, Elbe A-M, Hauge ML-T, Wikman JM. Late specialization: the key to success in centimeters, grams or seconds (cgs) sports. Scandinavian Journal of Medicine and Science in Sports 2011;21(6):e282-e290.

Ford PR, Williams AM. The developmental activities engaged in by elite youth soccer players who progressed to professional status compared to those who did not. Psychology of Sport and Exercise 2012;13(3):349-52.

Roca A, Williams AM, Ford PR. Developmental activities and the acquisition of superior anticipation and decision making in soccer players. Journal of Sports Sciences 2012;30(15):1643-52.

Barreiros A, Côté J, Fonseca AM. Training and psychosocial patterns during the early development of Portuguese national team athletes. High Ability Studies 2013;24(1):49-61.

Hardy L, Laing S, Barlow M, et al. A comparison of the biographies of GB serial medal and non-medalling Olympic athletes. London: UK Sport; 2013.

Güllich A. Selection, de-selection and progression in German football talent promotion. European Journal of Sport Science 2014;14(6):530-7.

Güllich A. Many roads lead to Rome – developmental paths to Olympic gold in men’s field hockey. European Journal of Sport Science 2014;14(8):763-71.

Güllich A, Emrich E. Considering long-term sustainability in the development of world class success. European Journal of Sport Science2014;14(Sup1):S383-97.

Huijgen BCH, Elferink-Gemser MT, Lemming KAPM, Visscher C. Multidimensional performance characteristics in selected and deselected talented soccer players. European Journal of Sport Science 2014;14(1):2-10.

Hornig M, Aust F, Güllich A. Practice and play in the development of the German top-level professional football players. European Journal of Sport Science 2016;16(1):96-105.

Drake D, Breslin G. Developmental activities and the acquisition of perceptual-cognitive expertise in international field hockey players. International Journal of Sports Science & Coaching 2017;13(5):636-42.

Güllich A, Kovar P, Zart S, Reimann A. Sport activities differentiating match-play improvement in elite youth footballers – a 2-year longitudinal study. Journal of Sports Sciences 2017;35(3):207-15.

Güllich A. International medallists’ and non-medallists’ developmental sport activities – a matched-pairs analysis. Journal of Sports Sciences 2017;35(23):2281-8.

Low JFL, Mohamad NI, Ong KB, Aziz SA, Abdullah MR, Maliki ABHM. The developmental pathways of Malaysian elite youth badminton players. Journal of Fundamental and Applied Sciences 2017;9:842-57.

Schroepf B, Lames M. Career patterns in German football youth national teams – a longitudinal study. International Journal of Sports Science & Coaching 2017;13(3):405-14.

Velentza E. A retrospective analysis of talent selection and progression within England’s rugby football union elite player performance pathway. Doctoral thesis, University of Chester, UK; 2017.

Hendry DT, Hodges NJ. Early majority engagement pathway best defines transition from youth to adult elite men’s soccer in the UK: A three time-point retrospective and prospective study. Psychology of Sport and Exercise 2018;36:81-9.

Güllich A. Sport-specific and non-specific practice of strong and weak responders in junior and senior elite athletics – a matched-pairs analysis. Journal of Sports Sciences 2018;36(19):2256-64.

Güllich A. “Macro-structure” of developmental participation histories and “micro-structure” of practice of German female world-class and national-class football players. Journal of Sports Sciences 2019;37(12):1347-55.

Hendry DT, Williams AM, Ford PR, Hodges NJ. Developmental activities and perceptions of challenge for national and varsity women soccer players in Canada. Psychology of Sport and Exercise 2019;43:210-8.

Güllich A, Cronauer R, Diehl J, Gard L, Miller C. Coach-assessed learning progress of youth soccer players correlates with earlier childhood practice in other sports. International Journal of Sports Science & Coaching 2020;15(3):285-96.

Noon MR, Eyre ELJ, Myers TD, Morris RO, Mundy PD. The influence of recruitment age and anthropometric and physical characteristics on the development pathway of English academy football players. International Journal of Sports Physiology and Performance 2020;16(2):199-207.

Dugdale JH, Sanders D, Myers T, Williams AM, Hunter AM. Progression from youth to professional soccer: A longitudinal study of successful and unsuccessful academy graduates. Scandinavian Journal of Medicine & Science in Sports 2021;31(S1):73-84.

Staff T, Gobet F, Parton A. Early specialization and critical periods in acquiring expertise: A comparison of traditional versus detection talent identification in Team GB cycling at London 2012. Journal of Motor Learning and Development 2021;9(2):296-312.

Andrew M, O’Brien RW, Ford PR, Causer J. Developmental activities of professional male British rugby-league players versus controls. Science and Medicine in Football 2022;6(3):381-88.

Garcia-Rubio J, García-Vallejo A, de los Ángeles Arenas-Pareja M, López-Sierra P, Ibánez SJ. From junior to elite in soccer: Exploring the relative age effect and talent selection in Spanish youth national teams. Children 2022;9(10):1543.

**Figure S1.** Effects of age at commencement of involvement in a talent promotion program on later performance in studies involving junior samples. Forest plot depicting the effect size (squares) and 95% confidence interval (error bars) of each study and the meta-analytic mean effect size (diamond).
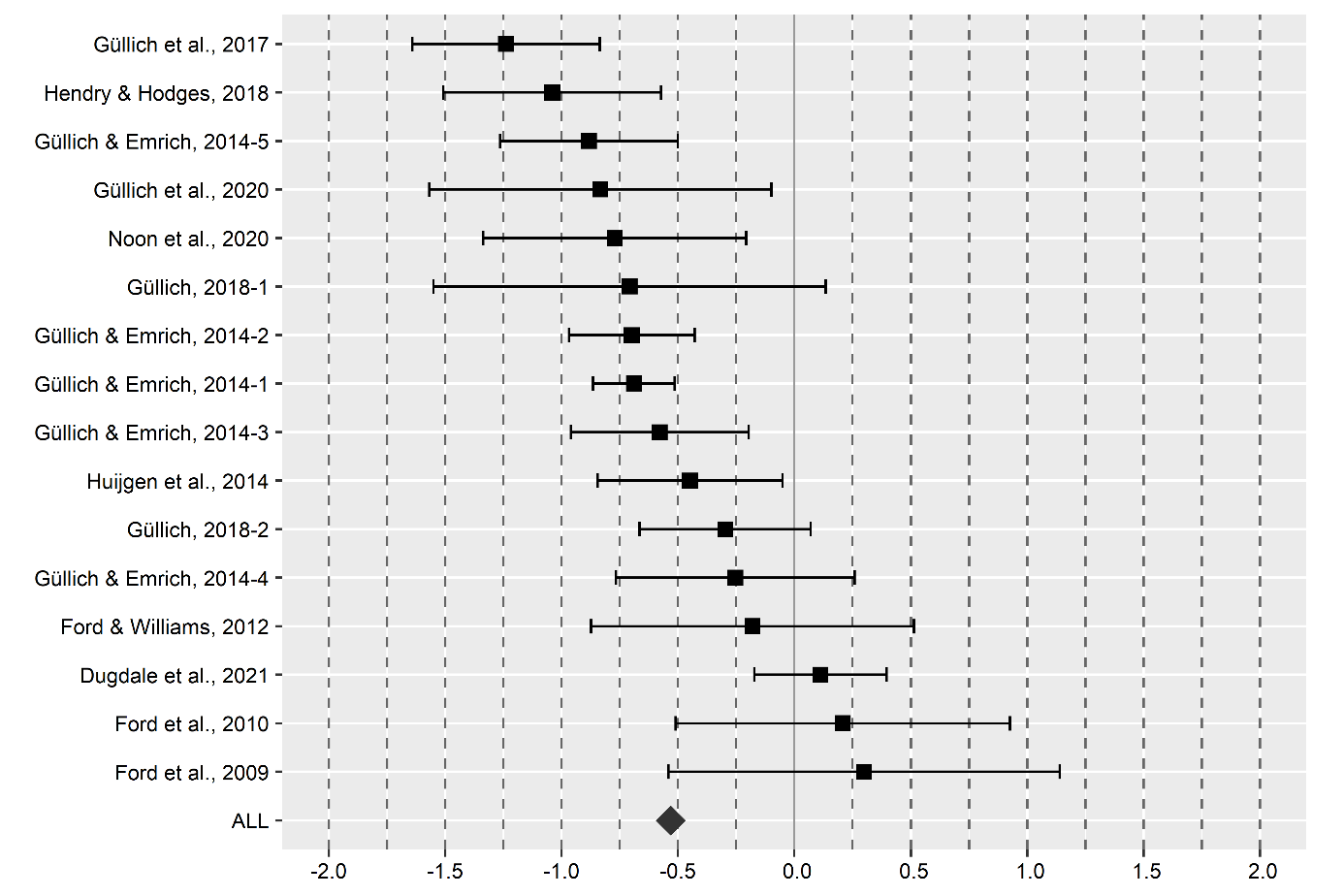


**Figure S2.** Effects of age at commencement of involvement in a talent promotion program on later performance in studies involving senior samples. Forest plot depicting the effect size (squares) and 95% confidence interval (error bars) of each study and the meta-analytic mean effect size (diamond).
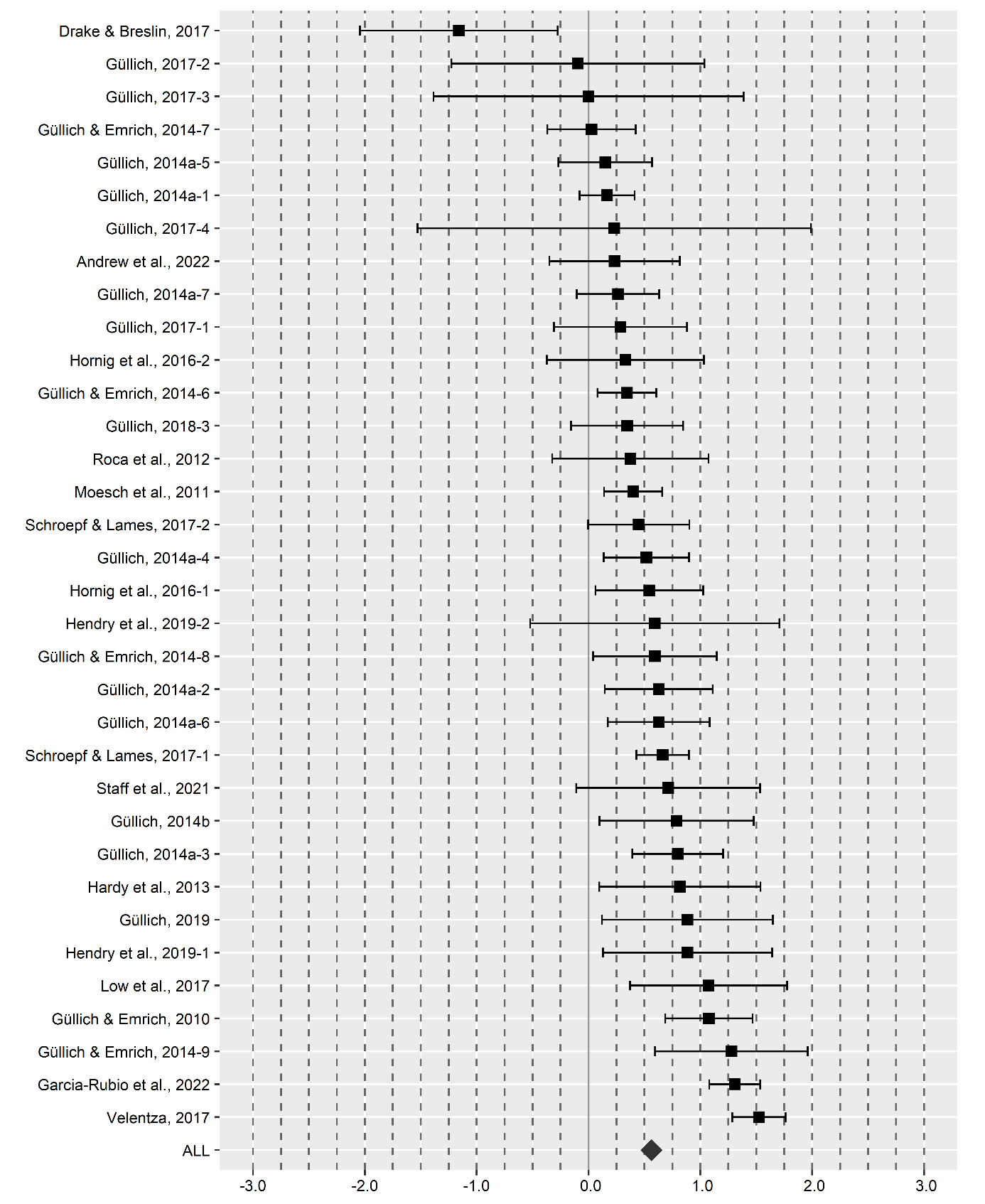


**Figure S3.** Funnel plot for the junior sample.
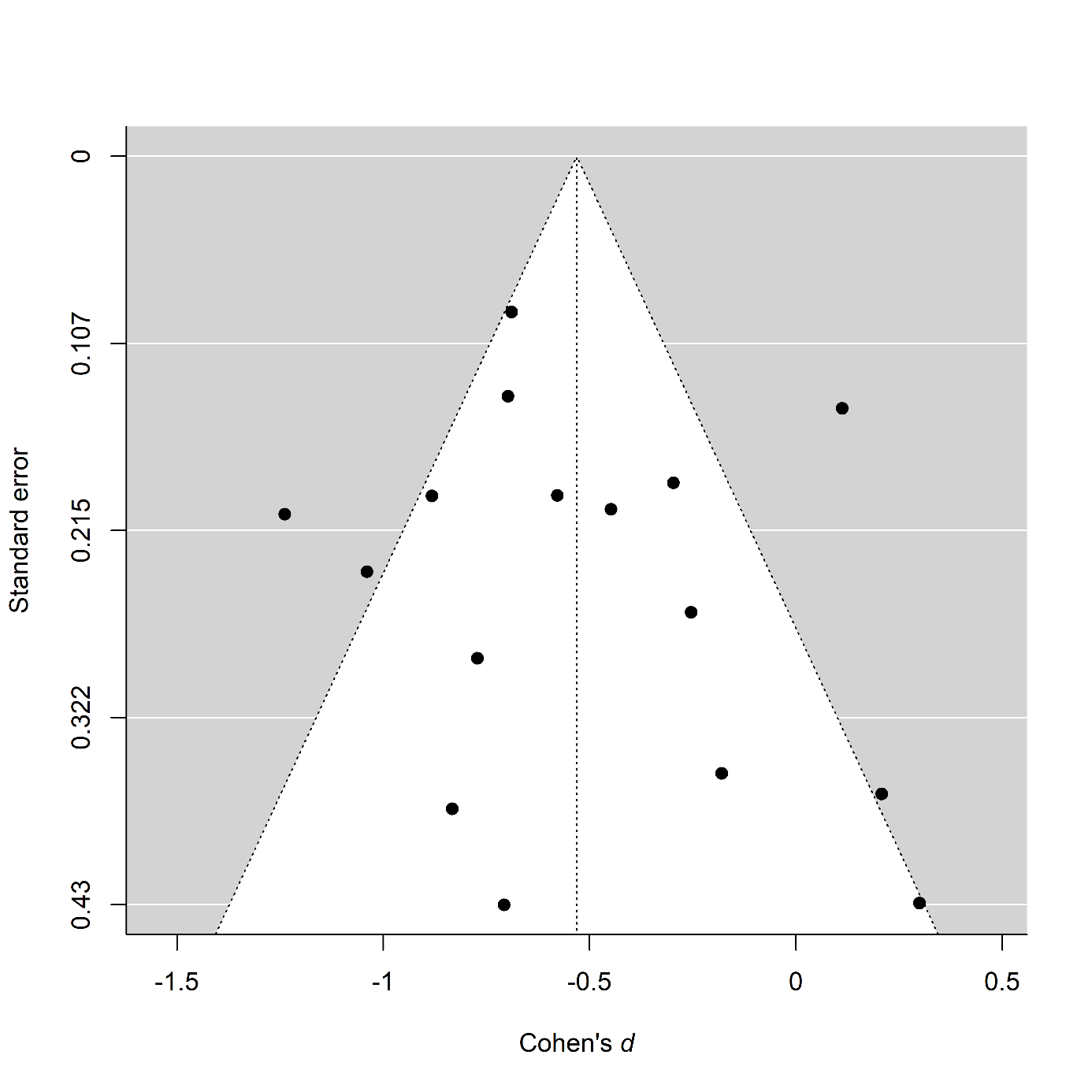


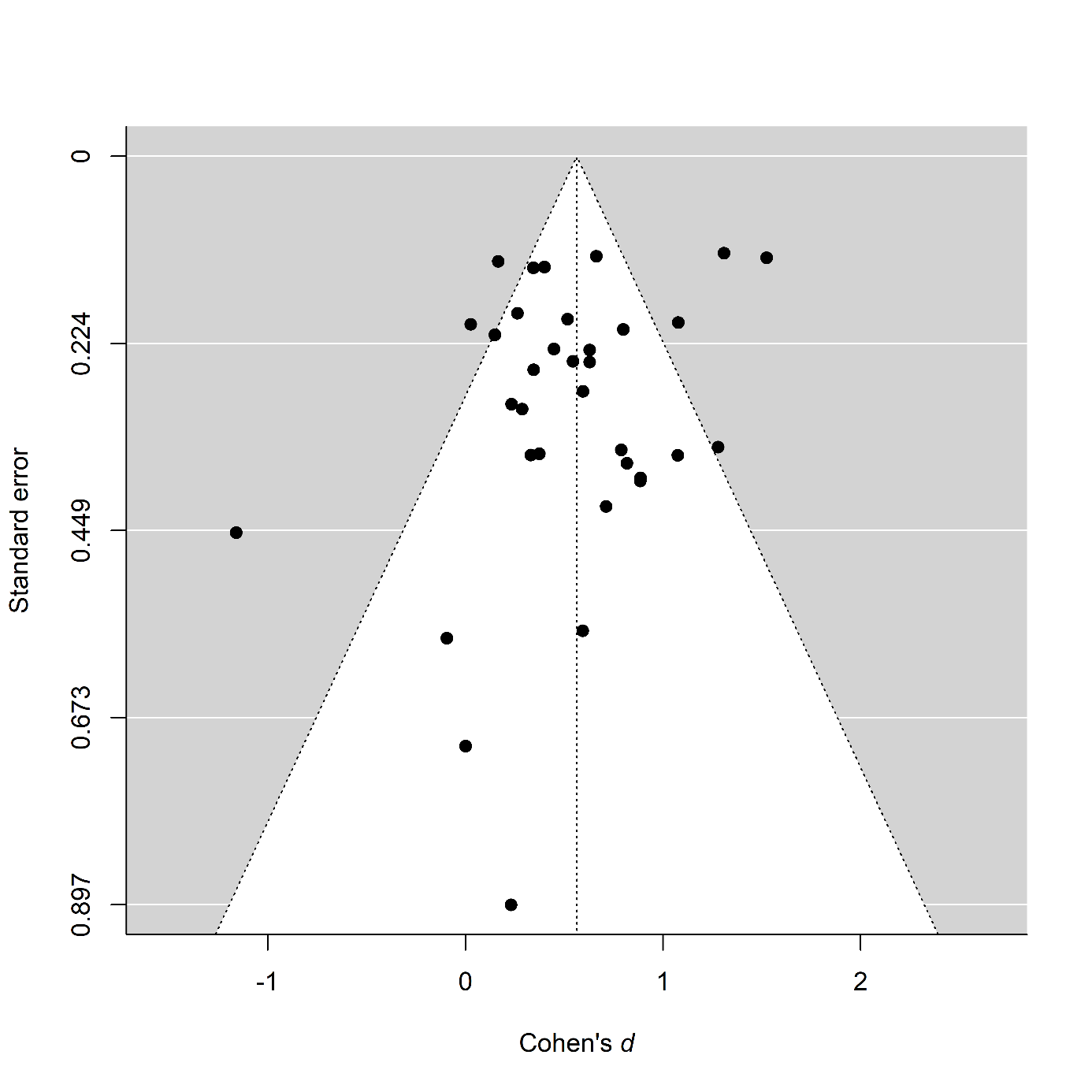


**Figure S4.** Funnel plot for the senior sample.

**Table S2.** MMAT assessment.
